# Adolescents’ Preferences, Experiences, and Views of 2 Online Programs for Anxiety and Depressive Symptoms: Qualitative Interview Study

**DOI:** 10.2196/84384

**Published:** 2026-08-18

**Authors:** Paula Kuberka, Madeleine Chilton, Katharine Pike, Roz Shafran, Lucy Yardley

**Affiliations:** 1 School of Psychological Science, Faculty of Health and Life Sciences, University of Bristol, 12a, Priory Road, Bristol, BS8 1TU, United Kingdom, 44 (0) 117 374 663; 2 Bristol Medical School, Centre for Academic Child Health, Faculty of Health and Life Sciences, University of Bristol, Bristol, United Kingdom; 3 Department of Paediatric Respiratory Medicine, Bristol Royal Hospital for Children, Bristol, United Kingdom; 4 Faculty of Population Health Sciences, UCL Great Ormond Street Institute of Child Health, London, United Kingdom; 5 School of Psychology, Faculty of Environmental and Life Sciences, University of Southampton, Southampton, United Kingdom

**Keywords:** Interviews, digital interventions, mental health interventions, DMHIs, mental health, young people, digital mental health interventions

## Abstract

**Background:**

Anxiety and depression are among the most prevalent mental health problems affecting adolescents. Digital mental health interventions have emerged as promising means of addressing these challenges. Understanding adolescents’ experiences and perspectives, and incorporating them into intervention design, can enhance their relevance, engagement, and effectiveness, ensuring interventions meet young people’s needs.

**Objective:**

This qualitative study aimed to identify how adolescents aged 16-19 years engaged with 2 distinct interventions (Bite Back [Black Dog Institute] and Project YES [Youth Empowerment & Support; Lab for Scalable Mental Health and Koko]). It explored (1) barriers and facilitators to engagement, (2) young people’s preferred design features and content, and (3) perceptions of their potential usefulness and helpfulness.

**Methods:**

Adolescents aged 16-19 years living in the United Kingdom were eligible to take part and were recruited through a university and a charity using convenience sampling. The final sample comprised 22 participants aged 18-19 years. They were asked to use Bite Back and Project YES for 30 minutes each over a 2-week period. Following this, 1-hour semistructured online interviews were conducted between January and March 2025. Anonymized transcripts were managed in NVivo (version 14; Lumivero) and analyzed using a pragmatic, inductive thematic analysis.

**Results:**

Seven themes were identified that captured young people’s views on factors influencing engagement, preferred design features and content, and the perceived usefulness of digital mental health interventions. Participants’ views were shaped by whether the content felt age-appropriate, culturally relevant, and relatable, and by how clear and transparent the interventions were. Interactive, visually appealing, and tailored features were generally preferred. Although most participants found Bite Back helpful, useful elements were identified in both interventions.

**Conclusions:**

This study contributes to the growing understanding of how young people engage with digital mental health interventions by identifying a set of interconnected factors that influence young people’s experiences. It highlights the importance of designing interventions that align with young people’s stage of life, culture, and experiences. Even small age differences appeared to influence how relevant the interventions felt to the individual. Furthermore, involving young people in co-design and ensuring clear, accessible communication may support trust and engagement, ultimately enhancing the benefits of such interventions.

## Introduction

### Background

The prevalence of mental health problems is estimated at almost 14% among individuals aged 15-19 years, representing the highest rate across all age groups up to 24 years [1]. According to the Global Burden of Disease study by the Institute for Health Metrics and Evaluation (IHME), anxiety and depression account for approximately 40% of these cases [2]. Adolescents with anxiety or depression often experience persistent sadness, irritability, low energy, or hopelessness [3-7], which can lead to social withdrawal, school avoidance, poor academic performance, and various health-related challenges [8-11]. When such difficulties occur during adolescence, a period of critical developmental changes, including identity formation, emotional regulation, and evolving social relationships [12-18], they can negatively shape future well-being. Without appropriate support, these challenges may persist into adult life, negatively affecting employment opportunities, interpersonal relationships, and overall quality of life [19-25].

Early intervention is essential, both to prevent the worsening of symptoms and to reach young people during a critical developmental stage, particularly as an increasing number of adolescents are experiencing anxiety and depression [26-30]. However, public mental health services frequently involve long waiting times [31,32]. In the United Kingdom at the end of 2023, only about one-third of adolescents with referrals to Child and Adolescent Mental Health Services (CAMHS) were able to access support within a year [33]. These delays highlight a gap between the growing demand for mental health services and the limited capacity of available professionals [34], creating barriers to accessing care. This problem can disproportionately affect individuals from ethnic minority groups or disadvantaged socioeconomic backgrounds, who may be more likely to experience such mental health difficulties [35,36]. Digital mental health interventions offer a promising avenue to bridge this gap, reduce inequalities, and deliver timely, accessible support to those who need it most.

The widespread use of digital devices, with as many as 96.5% of young people aged 12-17 years owning a smartphone in the United Kingdom [37], has paved the way for new approaches to delivering mental health support. Among these, digital interventions have emerged as valuable tools for helping young people manage anxiety, depression, and other mental health challenges [38]. Such tools can further offer the privacy, convenience, and flexibility that many young people seek [39-41], making mental health support more accessible. In addition to meeting the needs of young people, digital mental health interventions also offer a cost-effective approach [42] that could help alleviate the burden on mental health services by complementing existing care. By improving mental health literacy, these tools can also equip adolescents with skills required to better manage their symptoms and recognize when to seek professional help [43,44].

A wide range of digital mental health interventions has been developed in recent years, including those targeting anxiety and depression in adolescents [45-49]. These interventions are primarily delivered through mobile apps (eg, We Click [UNSW Sydney Brain Sciences] [50], CARE [Child and Adolescent Research in Emotion Lab] [51], and MoodHwb [Cardiff University] [52]), websites (eg, Grasping the Opportunity [53], An Enhanced Psychological Mindset Session [54], and MoodGYM [55]), or online platforms (eg, BetterHelp and Thriveworks). They draw on a variety of theoretical frameworks, such as positive psychology (eg, Bite Back [Black Dog Institute] [56]), mindfulness (eg, CARE [51], Tita [University of Turku] [57]), and growth mindset (eg, Project YES [Youth Empowerment & Support; Lab for Scalable Mental Health and Koko] [58] and Shamiri [Shamiri Institute] [59]), among others, but cognitive behavioral therapy (CBT; eg, Stressbusters [team from Manchester, The Institute of Psychiatry, London and Australia] [60], CATCH-IT [Competent Adulthood Transition with Cognitive Behavioral, Humanistic, and Interpersonal Training; University of Chicago] [61], and SPARX [The University of Auckland] [62]) remains the most commonly used framework. Digital interventions can address a broad spectrum of mental health challenges, such as eating disorders [63], posttraumatic stress symptoms [64], or sleep problems [65,66]. Some tools are intended for individuals with neurodevelopmental disorders [67] or chronic diseases [68], whereas others are aimed at the general population or at individuals experiencing elevated symptoms, as a form of prevention [69] or early support [70]. Some serve as a medium for delivering conventional therapy [71] or are designed to complement traditional approaches by offering additional resources or activities between sessions (eg, ChilledOut Online [Macquarie University] [72]), whereas others are intended to function independently, without direct support from a mental health professional [73].

Despite the growing number of such interventions, there remains a significant gap between their development, rigorous evaluation, and application in real-world settings. While many digital mental health interventions are publicly accessible, a substantial number have not undergone empirical evaluation [45,74,75], raising concerns about their effectiveness and safety. At the same time, few digital interventions targeting anxiety and depression that have been evaluated in research studies are actually implemented or widely accessible outside research settings [69,73,76]. Additionally, such interventions are still seldom developed with the involvement of the target population, which may limit their effectiveness [77-80]. This highlights a need for co-designed, practical interventions that are both evidence-based and accessible to adolescents beyond controlled research environments.

While assessing the effectiveness of an intervention is crucial, the success of digital mental health interventions also depends on how adolescents engage with them in real-world contexts. Even the most well-designed tools can fall short if they fail to capture users’ attention, meet their needs, or resonate with their lived experiences. Research has shown that adolescents often disengage from digital interventions due to issues such as limited interactivity, concerns about privacy, or unclear and unrelatable content [81-83]. Engagement itself is a complex construct that goes beyond simple metrics such as usage frequency or duration. It is important that user interactions with the intervention are meaningful and sufficient to support positive behavior change [84]. Therefore, investigating targeted user experiences with the interventions is essential to ensure that digital mental health interventions are effectively engaging and consequently beneficial for those experiencing mental health difficulties [85].

Existing qualitative evidence has highlighted that adolescents value interventions that feel relevant to their age, culture, and experiences [81-83,86,87]. Interactive and personalized features, ease of use, privacy, flexibility, and confidentiality further support engagement with interventions [82,83,87,88]. Concerns about privacy and perceptions that interventions are overly text-heavy or too clinical have been identified as barriers to engagement [82,89]. This supports the view that qualitative research plays a vital role in understanding adolescents’ experiences and perspectives on digital mental health interventions. User feedback obtained through qualitative methods helps ensure that interventions remain engaging, relevant, and responsive to the specific needs of adolescents [90]. Thus, understanding individual perspectives can provide deeper insights into what makes digital interventions acceptable, meaningful, and helpful, and further inform the refinement of intervention content and design beyond what quantitative measures alone can capture.

This qualitative study aimed to examine young people’s views and experiences of 2 distinct digital mental health interventions (Bite Back [56] and Project YES [58]) designed to address anxiety and depressive symptoms. Both interventions were used as reference points for adolescents and were not directly compared to one another because they differ considerably in design and content. The study explores key factors that influence how young people in the United Kingdom interact with these interventions, including specific features and content they may find beneficial or unhelpful, their preferences, and perceived usefulness and effectiveness. By examining the views of users who differ slightly in age and culture from the original target user population, this study seeks to contribute to our understanding of potential barriers to implementation and the need for adaptation of user-centered digital mental health interventions, supporting efforts to enhance their accessibility, relevance, and impact for a wider range of young people.

### Research Questions

This study was guided by the following research questions: (1) What are the barriers and facilitators to engagement with 2 distinct interventions (Bite Back and Project YES) among UK users aged 16-19 years? (2) What are young people’s preferred design features and content when comparing Bite Back and Project YES? (3) What are young people’s perceptions of the potential usefulness and helpfulness of Bite Back and Project YES for their mental health?

## Methods

### Ethics and Preregistration

Ethical approval was granted by the University of Bristol ethics board (reference number 21829), and the qualitative study protocol was registered on the Open Science Framework (OSF) [91]. This paper adhered to the Consolidated Criteria for Reporting Qualitative Research (COREQ) checklist [92].

### Participants

This qualitative study explored participants’ experiences of 2 distinct mental health interventions through 1-hour semistructured online interviews, analyzed using pragmatic thematic analysis. Adolescents aged 16-19 years living in the United Kingdom, with access to a smartphone or laptop and an internet connection, were eligible to take part in the study. Twenty-two adolescents aged 18-19 years were included in the final sample. Those who reported experiencing mental health symptoms that severely impacted their lives or who had ever been hospitalized due to mental health–related reasons were excluded from the study. These criteria were chosen primarily to complement the “Sleep Well” study, which recruited a nonclinical sample of mostly 16-18-year-olds. This age range was slightly expanded to include young people just above the age range in which Bite Back and Project YES had previously been evaluated, to explore how these interventions were experienced by slightly older adolescents.

### Procedure

Convenience sampling was implemented due to recruitment challenges, and as a result, purposive sampling based on age and ethnicity was not feasible. Most recruitment was facilitated through a university’s experimental-hours scheme, through which psychology students can earn course credit for participation in research. Five mental health charities working with adolescents were approached; only 1 agreed to assist with recruitment, while the remaining charities were unavailable. Interested adolescents first completed an online eligibility questionnaire. Those who met the inclusion criteria were invited to a brief video call with the researcher (via Microsoft Teams). During this call, which was not recorded, participants verified their nationality and age by presenting a valid photo ID. Eligible participants then received a Qualtrics (Qualtrics International Inc) link to the consent form and additional well-being, anxiety, and depression questionnaires. No relationship with participants was established prior to the start of the study. Participants received a £20 (£1=US $1.26 as of March 2025) Love2shop voucher or 2 experimental hours credits to compensate them for their time spent using the interventions and participating in the interview.

Participants provided consent and completed questionnaires (see Standardized Measures section) before they gained access to the interventions. They were then invited to engage with Bite Back and Project YES for a total of approximately 1 hour (30 minutes with each intervention) over a 2-week period, in the order they preferred. This duration was chosen to ensure that all participants had comparable exposure to both interventions while taking into account their academic commitments and the single 30-minute-session design of Project YES. As both interventions were freely available external programs, the actual time spent using the interventions was not formally tracked. Following this 2-week period, participants took part in a 1-hour, semistructured online interview that explored their views and preferences regarding these interventions.

### Sample Size Justification

To determine a sample size for this study, the concept of “information power” was applied [93]. This study incorporates relatively narrow aims and a fairly specific sample, focusing on UK adolescents aged 16-19 years and their views and experiences of 2 distinct digital mental health interventions. However, the use of convenience and purposive sampling to recruit participants may limit how targeted the sample could be. In addition, the study is not guided by a single established theoretical framework. Although the data were generated through 1-hour semistructured interviews that support rich interview data, both PK and MC were novice interviewers with some prior interviewing experience. Inductive thematic analysis is used to identify patterns across participants’ accounts. Taken together, these factors indicate that a sample size of 20 participants was considered adequate to provide the necessary information power to fully address the research questions.

### Interventions

Bite Back and Project YES were chosen through a systematic review [73] that was conducted as part of the “Sleep Well” study (ISRCTN14480620) [94], which focused on creating stepped care that aimed to help adolescents with their sleep, anxiety, and depressive symptoms. Within this review, both Bite Back and Project YES were identified as promising interventions based on effectiveness, risk of bias, and real-world applicability, and were the only freely available options in this category [73]. This evaluation was conducted independently, with none of the authors having any investment in either intervention.

#### Bite Back

Bite Back is a web-based positive psychological program developed at the Black Dog Institute in Australia. This interactive and self-guided 6-session program was designed to promote well-being and enhance resilience among young people aged between 12 and 18 years. Bite Back was not originally developed in collaboration with its intended user group. This intervention was openly accessible and has been evaluated through randomized controlled trials, demonstrating significant improvements in well-being, resilience, and life satisfaction as well as reductions in anxiety and depression symptoms in a nonclinical sample [56]. The Black Dog Institute in Australia offers further information about the Bite Back program on its website [95]. However, following completion of the study, the program itself is no longer accessible.

#### Project YES

Project YES is a freely available web-based program created by researchers at the Lab of Scalable Mental Health at Northwestern University, United States. This self-guided program comprises 4 single sessions, approximately 30 minutes in duration: Project Personality, the Action Brings Change (ABC) Project, Project CARE, and Project RISE. The sessions were based on the growth mindset, behavioral activation, self-compassion, and minority stress theories and were developed to support the mental health of young people aged 11-17 years. A recent randomized controlled trial demonstrated significant reductions in symptoms of anxiety and depression, hopelessness, and restrictive eating [58]. While the original version of the intervention was not developed with input from the target user group, subsequent adaptations of the intervention have been co-designed with young people [96]. More information about Project YES, which remains openly accessible, can be found on the Lab for Scalable Mental Health website [97].

### Data Collection

#### Overview

Data collection took place between January and March 2025 and was conducted by 2 female researchers (PK and MC). Interviews were automatically recorded via Microsoft Teams. Participants took part in a one-hour, one-to-one semistructured online interview guided by a topic guide (Multimedia Appendix 1), which was developed by the research team based on the research questions and content of both interventions. The topic guide was refined after the first few interviews to ensure clarity and relevance. Participants were initially asked about their experiences with Bite Back, followed by questions about Project YES. The topic guide comprised open-ended questions exploring participants’ experiences using the interventions, including engagement, usability, perceived helpfulness, and suggested improvements. If time permitted, an additional think-aloud component was included, in which participants were presented with screenshots of selected pages from the interventions and prompted to spontaneously verbalize their thoughts and impressions. This component aimed to elicit additional insights into specific elements of the interventions that participants might not have recalled spontaneously but could discuss when visually prompted. Recorded interviews were transcribed, and all identifiable information was removed to ensure participant anonymity. Following transcription, video and audio recordings were securely deleted.

#### Standardized Measures

Standardized questionnaires administered at baseline, before participants engaged with the interventions, along with demographic data (ie, age, gender, ethnicity, and parents’ highest level of education) were used to better understand and describe the study sample. This information allowed us to contextualize participants’ views and experiences of the interventions and to indicate the range of mental health presentations represented within the sample. The following measures were used:

##### Well-Being

Mental well-being was assessed using the Short Warwick-Edinburgh Mental Wellbeing Scale (SWEMWBS) [98,99]. The SWEMWBS is a short version of the Warwick-Edinburgh Mental Wellbeing Scale (WEMWBS). The SWEMWBS is a self-administered questionnaire consisting of 7 items that assess mental well-being using a 5-point Likert scale. Higher scores suggest more positive mental well-being. A reliability of α=.86 was reported for 16-19-year-olds [99].

##### Anxiety and Depression

Anxiety and depression symptoms were assessed using the Revised Children’s Anxiety and Depression Scale–25 (RCADS-25) [100]. The RCADS-25 is a short version of the RCADS. The RCADS-25 is a self-administered questionnaire consisting of 25 items that assess anxiety and depressive symptoms using a 4-point Likert scale (never, sometimes, often, and always). A higher score indicates more severe symptoms, with a cutoff point of 65 suggesting borderline clinical symptoms. A reliability of α=.86 was reported for 6- to 18-year-olds [101]. Although traditionally validated for individuals aged 8-18 years [100], emerging research has demonstrated its validity in 19-year-olds, supporting its appropriateness for this study population [102]. Thus, to maintain methodological consistency across the entire sample of 16- to 19-year-olds, this tool was selected as the sole measure.

### Data Analysis

An inductive thematic analysis [103] using a pragmatic epistemological approach was adopted, allowing the researcher to actively construct themes from qualitative data while ensuring that the findings reflected participants’ perspectives without using a preexisting framework. The themes remained closely grounded in participants’ views and experiences and, consistent with a pragmatic approach, focused on findings of practical relevance for intervention development and improvement. Data analysis was conducted independently by the primary researcher (PK), who had prior training in qualitative research methods. All authors were involved in discussing and refining the coding framework to enhance the credibility of the findings. The process followed the step-by-step approach to thematic analysis outlined by Braun and Clarke [103]. This involved familiarization with the transcripts, development of initial codes, and an iterative process of grouping and refining patterns across the dataset until the final themes were established. NVivo (Lumivero) software was used to manage the data [104].

As a researcher with a background in psychology and an interest in improving young people’s mental health, the primary researcher (PK) approached this study with a strong belief in the potential benefits of digital interventions. While PK had no affiliations with the digital mental health tools studied in this project, her personal interests may have influenced how she interpreted participants’ experiences, particularly if she unintentionally elicited or prioritized positive feedback. PK strove to balance her personal interest in improving mental health with an open-minded approach to understanding young people’s views and preferences. The potential influence of the researcher’s positionality, prior assumptions, and values about the topic was addressed through ongoing reflection, grounding interpretations in the interview data, and actively looking for examples that did not fit the developing patterns.

## Results

### Participants’ Characteristics

The results are based on 22 interviews. Most participants were White (n=16, 73%), 5 (23%) identified as Asian or Asian British, and one (5%) identified as having a mixed ethnic background. Participants were either 18 years old (n=8, 36%) or 19 years old (n=14, 64%). The majority were women (n=18, 82%), and most had no prior experience with online mental health interventions (n=15, 68%). Almost all participants had low-severity scores for depression (n=19, 86%), anxiety (n=20, 91%), and total RCADS-25 (n=19, 86%) scores, indicating that symptom levels were within the nonclinical range. Participants’ mean SWEMWBS score was 23.35 (SD 3.13), with higher scores indicating more positive mental well-being [105]. Detailed participant characteristics are presented in Table 1.

**Table 1.** Participants’ characteristics (N=22).

Characteristics	Values, n (%)	
Age (years)		
18	8 (36)	
19	14 (64)	
Gender		
Men	4 (18)	
Women	18 (82)	
Ethnicity		
White	16 (73)	
Asian or Asian British	5 (23)	
Mixed or Multiple ethnic groups	1 (5)	
Parents’ highest education level		
Secondary school up to 16 years	2 (9)	
Higher or secondary or further education (A-levels, BTEC^a^, etc)	4 (18)	
College or university	8 (36)	
Postgraduate degree	8 (36)	
Mean SWEMWBS^b^ score, mean (SD)	23.35 (3.13)	
RCADS-25^c^		
Depression		
Low severity	19 (86)	
Medium severity: borderline clinical threshold	1 (5)	
High severity: above clinical threshold	2 (9)	
Anxiety		
Low severity	20 (91)	
Medium severity: borderline clinical threshold	1 (5)	
High severity: above clinical threshold	1 (5)	
Total score		
Low severity	19 (86)	
Medium severity: borderline clinical threshold	2 (9)	
High severity: above clinical threshold	1 (5)	
Has previous experience with online mental health programs	7 (32)	
Device used to access Bite Back and Project YES^d^		
Laptop	21 (95)	
Phone	1 (5)	

a BTEC: Business and Technology Education Council.

b SWEMWBS: Short Warwick-Edinburgh Mental Wellbeing Scale.

c RCADS: Revised Children’s Anxiety and Depression Scale.

d YES: Youth Empowerment & Support.

### Theme 1: Relatability

**Figure 1.**
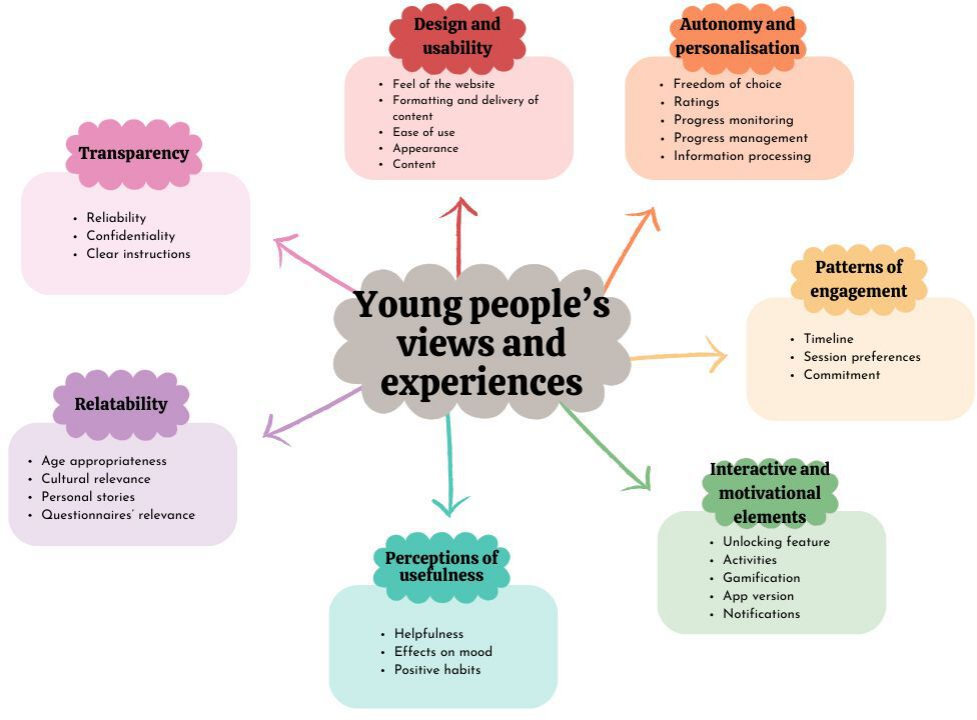
Visual representation of themes.

Relatability refers to whether participants felt that the interventions were relevant to their age, personal experiences, and the cultural references used within the content. Interventions targeted at younger audiences, those containing culturally mismatched information, or those failing to reflect participants’ lived experiences could affect young people’s engagement. See Figure 1 for an overview of all identified themes.

Participants frequently reflected on whether the tone, language, and content of the interventions felt appropriate for their stage of life. Many felt that some parts were targeted toward a younger audience, such as secondary school students. They reported that the information was overly simplified or that the language felt too basic. While most participants found this off-putting, others appreciated content that was accessible, clear, and welcoming to a range of age groups. A few described how more complex or mature content would have made it feel more suitable for them and encouraged them to continue using the intervention.

So just felt like it was aimed at someone much younger than me who might need help understanding certain terms.

[Participant 19, female, 19 years old, about Project YES]

I mean, I can imagine maybe people like my age or slightly older will probably be the age where people start thinking, oh, this is a bit childish, but for me personally, I liked it.

[Participant 15, female, 19 years old, About Project YES]

I think it might have been slightly younger than my age demographic, maybe by a few years, but that didn’t make it boring or anything. I still thought it was quite good.

[Participant 12, female, 19 years old, about Bite Back]

Cause of how simply they were trying to describe things like your strengths or like your social connections, […] like defining everything and then that’s what made it seem like less age appropriate.

[Participant 19, female, 19 years old, about Bite Back]

Familiarity with cultural references also influenced how relatable participants found the interventions. Some individuals found it difficult to connect with content that included Australian or US-based brands, examples, or language rather than references that felt familiar within a UK context. However, not all participants mentioned such cultural discrepancies.

It’s very I think Australian, not British. And lots of the token things where you could choose which brands you liked. I don’t think I recognized any of the brands.

[Participant 11, female, 18 years old, about Bite Back]

Like it’s targeted toward, like, an American audience like it asked: Are you in high school? […] Are you this and I’m not. So, it did obviously feel a bit […] distance from that!? It’s like I did feel like it wasn’t made for like a tailored British audience.

[Participant 8, male, 19 years old, about Project YES]

Some participants indicated that other people’s experiences made the intervention feel more personal. However, others pointed out that, although the stories felt like a valuable component of the intervention, they became less impactful when unrelated to their current stage of life.

I think it makes it a bit more specific, and it also gives you actual real-world examples of it instead of just concepts that are floating about. […] But then because there was so many of them and a lot of them were very similar, they were all in like kind of a similar setting, similar situations that got a bit repetitive and boring

[Participant 1, male, 18 years old, about the personal stories in Project YES]

Some stories I probably connected to, and I understood, whereas other ones that I was less connected to, I got bored so […]. Yeah, but the relevance of the story to my own experiences was a big factor in whether I felt engaged with it

[Participant 8, male, 19 years old, about Project YES]

Maybe if they used like examples that were a bit more relevant to someone who’s older.

[Participant 9, female, 19 years old, about both interventions]

Some participants queried the inclusion of questionnaires at the beginning of the interventions. They expressed uncertainty about their purpose and how, or whether, the responses were used. For these participants, the questionnaires felt unnecessary and uninformative. Although questionnaires in both interventions were not used for tailoring, Bite Back users received some feedback depending on their scores, which some participants valued. However, most participants did not question the relevance of these questionnaires.

It was sort of like you just did the quiz at the start and then it was not mentioned again. Yeah, 'cause, I think it would provide more relevance to you actually having to do it at the start rather than just doing the quiz and then like forgetting about it

[Participant 12, female, 19 years old, about Project YES]

One part I liked about the Bite Back one was that they did give you a quiz at first and then gave you a score. So, I think Project YES could probably do something like that. I liked it because it kind of gave me an idea about like how I was about like each specific topic and then from that, I can like move on to the activities and the videos

[Participant 3, female, 18 years old, about both interventions]

### Theme 2: Transparency

Transparency relates to the clarity of instructions, openness about participants’ data use, and the reliability of the interventions’ content. A lack of clarity around what the tasks involved or where participants’ information was going could affect adolescents’ willingness to fully engage with the interventions.

A few participants highlighted that the statistical content provided in Project YES made the information feel more legitimate and helpful. This was the only aspect of either intervention that participants specifically identified as contributing to a sense of reliability.

I kind of like the statistics. I think ’cause it made it kind of seem more legitimate when they added all of that.

[Participant 9, female, 19 years old, about Project YES]

Like I think it can make you feel a lot like, […] say if you’re doing this and you think you’re a minority or you think you’re like really strange or something and then you see statistics that like, oh, most people think this or most people are in the same boat. I do think it makes you feel more like you’re not just a weirdo

[Participant 1, male, 18 years old, about Project YES]

Several participants expressed uncertainty about what would happen to the information they shared. Some felt uneasy when the intervention did not clearly explain how their responses would be used or stored. This lack of clarity could lead to discomfort, which might act as a potential barrier to engagement.

During the session, it didn’t really explicitly say where the information was going, and that was quite off putting

[Participant 11, female, 18 years old, about Project YES]

I had to put like I don’t know like my post code and all these other information. Why does a mental health intervention need me to make an account and put my address and my phone number in. So yeah, it put me off

[Participant 8, male, 19 years old, about Bite Back]

A few participants suggested that clearer guidance on how to begin and navigate the interventions would be helpful. They described moments of uncertainty about how to start tasks or move through sessions. These suggestions highlight the importance of having explicit, well-structured instructions to support smoother engagement.

[What could have made it easier to use?] Perhaps making it more clear like that is going to be over a few weeks and what it’s actually trying to do and how it’s going to help you.

[Participant 14, female, 19 years old, about Bite Back]

Perhaps like for example with the name thing again, like it might have been a bit easier if there was like instructions at the top saying like oh, only put one name.

[Participant 13, female, 18 years old, about Bite Back]

### Theme 3: Design and Usability

Design and usability refer to the structure, presentation, and navigation of the interventions. Adolescents commented on the visual appeal, clarity of layout, and how easy it was to use and understand the content. A user-friendly layout, inviting appearance, and clarity of delivery could influence how adolescents interacted with the interventions.

Participants addressed the tone and presentation of the interventions. Bite Back was generally described as inviting, welcoming, and engaging, while Project YES was occasionally seen as more like a formal survey or test. Some participants who described feeling this way about Project YES also noted that it made them feel bored. A welcoming and well-structured website may influence the level of user participation.

I felt quite a nice, like, warm, like, welcoming feel to it.

[Participant 13, female, 18 years old, about Bite Back]

I felt like I was just doing a survey, but how it was like structured, it felt like a test rather.

[Participant 19, female, 19 years old, about Project YES]

Participants commented on the formatting and structure of the interventions. Bite Back was described by some as professional and well-designed. A few participants noted that the weekly format and content organized in smaller chunks made the sessions feel shorter and easier to follow. A few also noted that the weekly format made it easy to forget about the intervention. One participant said they would have preferred that the content take up more space on the screen.

It wasn’t just like reading words, there was audios and interactive activities with the typing and everything.

[Participant 12, female, 19 years old, about Bite Back]

It had the weeks, so it was like the sections that you could do. I thought it was nice because it organized them into little chunks.

[Participant 4, female, 19 years old, about Bite Back]

I think it was quite good. I quite like the reading because it wasn’t like that much to read. While sometimes you might find yourself like having to read like an entire page and then you get lost. I liked how concise the information was.

[Participant 19, female, 19 years old, about Bite Back]

In contrast, participants described Project YES as having a simpler design. Some said the platform was not user-friendly and mentioned that scrolling through pages was a long process. The presentation was occasionally compared to a textbook or “Wikipedia” page, and some felt that the large number of slides with minimal information elongated the experience. Suggestions included reducing the amount of text and making it more interactive.

I feel like it’s just plain black and white, it has like a little photo. It gives very for me textbooky or like Wikipedia vibe.

[Participant 9, female, 19 years old, about Project YES]

I think it was quite focused on like questionnaires and things like that. I think it would have been, it could have been more interactive.

[Participant 12, female, 19 years old, about Project YES]

Both interventions were described by many participants as easy to use. Bite Back was reported as having clear instructions, a straightforward interface, and short, accessible activities. Some said it was easy to start using and to return to the page if they had to leave. However, a few participants mentioned that logging back in required effort, and one noted that signing up took time. Others described needing to refresh the website or listen again to audio to be able to move to the next week’s session when the connection was lost.

I thought the interface, was very easy to use. It wasn’t too, like overwhelming with information or anything like that.

[Participant 2, female, 18 years old, about Bite Back]

It wouldn’t let me click on to the next page like, even though I listened to the whole audio, I clicked to go to the next page and it wouldn’t let me on. So I had to listen to audio again.

[Participant 8, male, 19 years old, about Bite Back]

Several participants said Project YES was easy to start using because no login was required, and some described the interface as clear and the navigation as straightforward. Others, however, said it was less easy to use, with one reporting difficulty navigating through the sessions and another stating it took more effort to use than Bite Back. A few participants also mentioned that getting through the sessions was a long process. Difficulties with logging in or navigating could create barriers to participants’ engagement with the interventions.

I think it was easy. I don’t remember having to go through the same login process as I did with Bite Back, so that was good.

[Participant 11, female, 18 years old, about Project YES]

It was quite easy once you like started doing the session. So then it’s kind of like, there’s not much to like, accidentally click on. It’s like you just go next, next.

[Participant 19, female, 19 years old, about Project YES]

Visual elements such as colors, fonts, and images contributed to how appealing and approachable the interventions felt. Participants described the colors and variety of fonts as uplifting and inviting. Images, especially cartoons or symbolic visuals, were positively received when they complemented the session content.

I like the colors, it just makes it seem less depressing.

[Participant 19, female, 19 years old, about Bite Back]

I think Project Yes doesn’t really have that much kind of like colors or things that you know really attract a person. The visual elements are really important.

[Participant 6, female, 18 years old, about Project YES]

Participants expressed mixed views on the content of both interventions. Some described the content in both interventions as familiar or “common sense,” reporting that it often felt like a repetition of information they already knew. While a few participants found this repetitive or not educational, others appreciated the opportunity to revisit known material and considered it a helpful reminder. Several participants mentioned that the concepts were explained using simple language. For some, this contributed to perceptions that the content was overly simplified or infantilizing. However, there were some participants who valued the inclusion of psychological concepts that were explained simply or described the content as scientific.

I thought it just covered like the generic, like mental health advice type of stuff you would see on like YouTube or in assemblies in school.

[Participant 9, female, 19 years old, about Bite Back]

It’s still very useful information. It, I believe, would help a younger individual because when we’re younger we see things a lot differently and I believe that it would help them.

[Participant 22, female, 18 years old, about Project YES]

### Theme 4: Autonomy and Personalization

Autonomy and personalization refer to features that allow participants the freedom of choice, control, and flexibility within the interventions. Participants referred to existing features such as selecting sessions, receiving feedback, and viewing progress. Suggestions to save progress and comments on format preferences reflected a desire for greater control over when and how they interact with the material.

Most participants liked or were positively surprised by the feature that allowed them to choose which session to work on in Project YES. Some said that having this choice made the intervention feel more tailored to them.

It felt like a more personalized approach rather than just forcing everyone to do the same activity, you can pick whichever one you think suits you.

[Participant 12, female, 19 years old, about the choice of sessions in Project YES]

You can choose whether you want to do this one or this one. It depends on how you’re feeling. I like that a lot.

[Participant 8, male, 19 years old, about Project YES]

Participants described the use of scores and ratings in Bite Back as a reflective and engaging feature. Several reported that receiving a score helped them learn more about themselves, visualize their progress, or feel more motivated to continue. Questions were generally viewed as clear, age-appropriate, and easy to use. However, one participant noted that receiving a low score could potentially be demotivating for some users.

Like this it can maybe put someone down and not want to carry on, because if they answer no, I’m not thankful. I’m not thankful and they see 5 out of 25 as the score. They’re not particularly going to be happy with that.

[Participant 14, female, 19 years old, about Bite Back]

It kind of tells you how you’re doing in that sort of environment. Like for example, I was doing really good in like the friends one but I’m not really good at like, the mindfulness one. […] I like how that was like, personalized.

[Participant 8, male, 19 years old, about Bite Back]

Participants referred to Bite Back’s progress bar as a feature that helped them track their progress through a session. While not all participants mentioned it, those who did described it as helpful and motivating. Some said it gave a clear sense of progression and an end point to work toward. A few mentioned missing a similar feature in Project YES.

It’s nice to know how far through you are, it’s good motivation to keep going.

[Participant 11, female, 18 years old, about the progress bar in Bite Back]

I feel like it’s always nice to see when you’re making progress on something, even if it’s like small. So I thought that was quite nice to see because I was like, oh, look at me, I’m doing well.

[Participant 13, female, 18 years old, about Bite Back]

Some participants suggested that the ability to save their responses to tasks could improve their experience with the interventions. A few mentioned that it would be helpful to revisit previous answers as a way to reflect on or recall how they felt at a particular time.

I feel like I would have also liked if they like, sent it to you. Obviously if they kept anonymous, but it would just be nice to have it there saved.

[Participant 9, female, 19 years old, about the activities in Project YES]

I think it would have been maybe nice to just have it like saved somewhere so you have the option of going back, because then maybe if you were […] having like a really bad day, you could go back to it and be like OK, […] what was I?! I’m having a bad day today. What was I grateful for at that time. It can give you a nice little reminder.

[Participant 1, male, 18 years old, about the activities in Bite Back]

Participants expressed varied preferences for how information was delivered. Some preferred reading text over listening to audio or watching videos, noting that reading was quicker, easier to process, or better suited to their learning style. However, many participants appreciated having multiple formats available, such as audio, video, and text, and described this as helpful or accessible. The ability to revisit the same content in different formats, such as having text that repeated video content, was seen by some as useful, while others felt that such repetition should be avoided. Several participants mentioned that bullet points would make the text easier to follow, particularly if they missed parts of a video.

A text that came after, it just repeated the video, and I would much prefer to skim over the text than watch the video.

[Participant 11, female, 18 years old, about Bite Back]

I quite liked the little audios that were there that you could like, listen to as you’re reading along, was just easier.

[Participant 9, female, 19 years old, about Project YES]

### Theme 5: Patterns of Engagement

Patterns of engagement describe how participants interacted with the interventions over time. This theme includes when and how they used the content, which sessions they continued with or disengaged from, and how they perceived the interventions in terms of accessibility and commitment.

Participants described varying patterns in when and how long they engaged with the interventions. Some completed multiple sessions in one sitting, often influenced by having free time or wanting a distraction. Others reported using the interventions in response to specific emotional states, such as feeling stressed, down, or frustrated. The time spent on each session ranged from 20 to 40 minutes, which many participants viewed as an appropriate or optimal duration. A few appreciated the short and manageable structure of both interventions.

When the website was used] It was in the morning just to sort of set myself up for the day. By practicing something different, because that’s […] when my anxiety peaks. And so it was nice to have, like, a routine in the morning with something to do, to practice, just to sort of like, take the time for myself and chill out.

[Participant 2, female, 18 years old, about Bite Back]

Used the website] when I had nothing else to do, like it wasn’t really like something I picked up to do because I needed like a break.

[Participant 19, female, 19 years old, about Bite Back]

Participants noted that not all sessions were equally appealing, and some expressed a desire for more flexibility in how they moved through the interventions, as this influenced how far they progressed. Several mentioned wanting to skip sessions they found less engaging, particularly the mindfulness session in Bite Back, which was sometimes seen as requiring a specific mindset. A few participants reported stopping their use of the intervention when they reached this session, suggesting that being unable to move past less preferred content could discourage continued engagement.

I wanted to unlock Level 3 without having to do 10 minutes of meditation.

[Participant 11, female, 18 years old, about Bite Back]

Like they’re forced to fill in the sentences to move on.

[Participant 3, female, 18 years old, about Project YES]

A few participants described the interventions as low-commitment or a more accessible alternative to more formal support services, such as university counseling.

It’s much like lower commitment than trying to like get a hold of the university counseling service.

[Participant 11, female, 18 years old, about Bite Back]

It’s an easy thing to get into because it’s like not asking for huge commitments. You can just do one week if you want or you can do like multiple of the like the week modules. Which I think’s good.

[Participant 7, female, 19 years old, about Bite Back]

### Theme 6: Interactive and Motivational Elements

Interactive and motivational elements refer to intervention features that could encourage engagement and support a more active experience. This theme includes both the elements present in Bite Back and Project YES, as well as participants’ suggestions for enhancing interactivity and motivation. It encompasses subthemes such as the unlocking feature, activities, gamification, an app version, and notifications.

Participants expressed mixed views about the unlocking structure of sessions in Bite Back. Several described it as motivating, noting that progressing through the weeks felt like achieving small goals or even like playing a game. Some said it encouraged consistency and ensured they moved through the content gradually. However, a few participants preferred having all sessions available from the start so they could choose content most relevant to them. One participant noted that the unlocking structure shortened their time spent using the intervention.

I think I’d rather have access to all of the weeks because there were certain weeks that for me I quite liked like the I think there was mindfulness, one which was really good, but then maybe on a specific day, I didn’t really want to be doing the, say, gratitude one. If I was in a specific mood and I wasn’t really feeling that. So I think it would be nice if they all were. Maybe if you were encouraged to do them in order, but they weren’t locked.

[Participant 4, female, 19 years old, about Bite Back]

I did enjoy the whole concept of unlocking like a new week or like you couldn’t start one before. I felt like I was almost playing a game, but then it was like a fun game, like AI. Well, I’ve done this now and now I’ve been rewarded with next week’s and like a star.

[Participant 13, female, 18 years old, about Bite Back]

Participants expressed a range of views about the activities included in both interventions. Many found the interactive components engaging and reflective, with some describing them as encouraging deeper thinking about personal experiences or relationships. Activities involving giving advice were often highlighted as particularly engaging and meaningful, though a few participants noted that these tasks could feel irrelevant when based on scenarios they had not personally experienced. In Bite Back, short, varied activities were generally well received, with participants appreciating their manageable length and real-life applicability. In contrast, some participants felt the tasks in Project YES became repetitive, overly focused on writing, or difficult to complete. Suggestions included incorporating a greater variety of scenarios and making the tasks more general so that more people could relate to them.

So the activities were all very like, made me reflect and stuff like that.

[Participant 9, female, 19 years old, about Bite Back]

I quite like the thing about […] giving the advice cause although it was effortful, I feel like it’s almost like encouraging you to give advice to yourself if you were in that situation. So, I think that did stand out.

[Participant 12, female, 19 years old, about Project YES]

Some participants suggested that incorporating game-like features could make the interventions more engaging. One participant said that receiving a reward at the end of a session would feel motivating and similar to playing a game.

Maybe including something like a game where you do the interventions and then it progresses through like a game sort of thing and then you get rewarded at the end or something like that.

[Participant 12, female, 19 years old, about Bite Back]

Several participants suggested that having access to the interventions through a dedicated mobile app would improve convenience and usability. An app-based format was seen as a way to encourage more consistent use by reducing barriers such as having to log in via a browser or remembering to return to the website after taking a break.

An app form would be quite interesting. I think an app like if it’s just on your phone, could be more accessible to people.

[Participant 9, female, 19 years old, about Bite Back]

I think an app would be convenient so that I don’t have to keep going back on my laptop and then opening it, and I can just use it on my phone.

[Participant 3, female, 18 years old, about Bite Back]

Most participants identified notifications as a potentially helpful feature for supporting consistent engagement with the interventions. They suggested that reminders could encourage them to return to the platform regularly, helping them stay on track with sessions and avoid forgetting about the program over time.

Like they said to have those activities to like repeat them every week or every day, if they had like a little maybe reminder like notification option, I think that could be useful.

[Participant 9, female, 19 years old, about challenges in Bite Back]

Just notifications on your phone and then it will remind you because obviously we’re all human. Like we’re forgetful, people are going to forget and stuff.

[Participant 15, female, 19 years old, about Project YES]

### Theme 7: Perceptions of Usefulness

Perceptions of usefulness capture participants’ reflections on how the interventions supported their mental health and well-being. Adolescents discussed overall helpfulness, impact on mood, and how the interventions encouraged positive habits.

Most participants perceived Bite Back as more helpful than Project YES. Many said they would be willing to recommend it to younger friends or family members. However, there were some participants who found Project YES beneficial, while others felt that both interventions offered useful elements. A few expressed a desire to revisit Bite Back to explore additional sessions.

It’s kind of like an easier way of like grounding yourself compared to like other forms.

[Participant 19, female, 19 years old, about Bite Back]

It’s easier to like actively engage in, whereas the other one [Project YES] was just like […] reading quite a few stories and things. Whereas the other one [Bite Back] was more like relating it to your own life, if that makes sense. So that was more engaging.

[Participant 5, female, 19 years old, about Bite Back]

[Which platform did you find more helpful?] Overall Project YES, but I think some of the activities in Bite Back were really good. I think if you could transport them then Project Yes would be perfect.

[Participant 4, female, 19 years old, about Project YES]

Several participants mentioned that using Bite Back made them feel calmer and more positive afterward and indicated that the intervention was a good starting point for improving their well-being. Some also reported positive changes after using Project YES.

There was definitely a more sense of like calm, I guess.

[Participant 19, female, 19 years old, about Bite Back]

Yes, after I used it, I did feel like a sense of accomplishment because of giving the advice and helping others.

[Participant 6, female, 18 years old, about Project YES]

When recommending Bite Back to others, some participants emphasized its potential to support the development of positive daily habits, even though not all reported forming these habits themselves.

I probably just tell them that it’s good for keeping those like positive habits.

[Participant 9, female, 19 years old, when recommending Bite Back to a friend]

## Discussion

### Principal Findings

This qualitative study used 2 distinct and widely available digital mental health platforms (Bite Back and Project YES) to help understand what supports or hinders young people’s engagement, what design features they prefer, and how useful they find these interventions. Participants frequently reflected on whether the tone, language, and content of the interventions felt age-appropriate, culturally relevant, and connected to their personal experiences. Clarity of instructions, transparency around data use, ease of navigation, and flexibility in how the interventions could be used also appeared to influence engagement. Participants generally preferred features that were interactive, visually engaging, and personalized, and many valued being able to track progress or choose content. Bite Back was often described as helpful, although some participants also found Project YES beneficial and identified useful elements in both interventions. These findings highlight a number of factors that may influence how young people engage with and perceive digital mental health interventions.

Facilitators and barriers to engagement were shaped by multiple, interconnected factors. Relatability and transparency were among these, with participants preferring interventions that used culturally relevant, age-appropriate content that aligned with their stage of life, supporting previous research emphasizing the value of content that resonates with adolescents’ lived experiences [81-83,86,87]. It is important to note that although participants in this qualitative study were only slightly older than the intended user age group, many described the content and design of both interventions as age-inappropriate. This indicates that even small age differences may affect perceived relevance and adds to existing research showing that developmental stage matters in digital mental health intervention design [106,107]. This highlights the need to involve young people across different adolescent age ranges in intervention development, especially when designing interventions targeting broader age groups. Although tailoring content to very narrow age bands may enhance relevance, this is not always practical in real-world settings. A more feasible approach may involve developing interventions that are adaptable to different developmental stages. In the future, artificial intelligence may offer scalable ways to personalize content dynamically based on age, preferences, or individual needs. Transparency regarding the purpose of the interventions, confidentiality, and instructions was of particular importance to participants, supporting previous research identifying transparency as a key factor in building trust and engagement [81]. Participants expressed a need for clear explanations on what happens to their data, how they could benefit from such interventions, and the purpose of certain tasks. The way transparency is communicated could matter, while clear information might foster trust, overly detailed or complex explanations might be overwhelming and may conversely hinder engagement. This suggests that interventions should balance providing essential information in a clear and accessible manner while avoiding information overload, ensuring young people feel informed and confident in engaging with digital mental health interventions.

Autonomy and personalization, patterns of engagement, and interactive and motivational elements were also identified as important factors influencing engagement, further aligning with previous research demonstrating their role in supporting young people’s use of digital mental health interventions. Participants valued the ability to track their progress and choose the order or type of content they engaged with [82,87], supporting a sense of control and relevance. Patterns of engagement indicated a general preference for shorter sessions [82,83,87], with some participants expressing that digital mental health interventions felt less demanding and required less commitment than student counseling services. Most participants engaged with the interventions only once without returning, describing differences in motivation for when to use them, which was often shaped by their mood, available time, or daily routines. This highlights the need for interventions to allow flexible use that aligns with adolescents’ lives, in line with broader research showing that ease of access and fit with everyday routines can influence how young people engage with digital mental health platforms [82,83,86,87,108]. Some participants suggested that the existing feature of session unlocking could help sustain motivation and recommended additional elements such as gamification, a mobile app version, or notification reminders to further encourage uptake and engagement. However, preferences varied, with some participants expressing the need for notifications as reminders and an app version for ease of access. Some participants preferred having all sessions unlocked to be able to choose those that were most appropriate for them, which is consistent with findings from the broader literature [82]. This reflects the importance of offering customizable interactive features to cater to individual preferences.

Participants preferred design features and content that offered clear and simple information, were easy to navigate, and featured a colorful, pleasing appearance. The ability to choose the order of sessions and to track progress was valued [82,87], which might have provided a sense of control and accomplishment. Informative content was appreciated, particularly when it was available in various formats, such as text, audio, or video, allowing participants to engage with material in ways that suited their preferences. These findings indicate that visually pleasing design, flexible use, and access to content in multiple formats are valued by young people and may further influence their engagement with digital mental health interventions. This is consistent with broader mHealth design research, which highlights navigation, presentation of information, personalization, and aesthetics as features associated with stronger user engagement [109].

Participants described the interventions as grounding and as a good starting point for improving well-being, with some expressing willingness to revisit them in the future. Participants’ views on the usefulness of the interventions suggest that even when an intervention includes therapeutic, evidence-based content, this may not be enough on its own for young people to find it helpful. This aligns with previous qualitative research highlighting the importance of perceived usefulness in sustaining young people’s engagement with digital mental health interventions [81,108].

All themes identified in this study are interconnected and collectively may shape how young people engage with digital mental health interventions and what they perceive as helpful within them. Design features and content preferences may influence whether young people find interventions appealing, practical, and worth returning to, which in turn may affect how much they benefit from using them. This highlights the importance of involving the target group in intervention development to ensure that interventions reflect young people’s needs and preferences. Overall, the findings support a broader body of research suggesting that helpfulness and engagement are shaped not only by intervention content, but also by whether digital mental health tools feel relevant, easy to use, and trustworthy to young people in practice [110].

### Strengths and Limitations

The use of a think-aloud component in an interview setting is a relatively novel approach and was generally perceived as a helpful technique for aiding recall and encouraging reflection on the interventions. Most participants noted that the prompts made it easier to remember and discuss specific aspects they might have otherwise overlooked, which could have enhanced the depth of feedback they provided. However, some participants found it challenging, suggesting that while think-aloud can enrich data collection, it should be introduced carefully to ensure participants feel comfortable sharing their perspectives.

Convenience sampling resulted in a participant pool that primarily consisted of university-educated individuals, including psychology students, who did not have elevated anxiety or depression and who might have greater familiarity with mental health concepts and digital tools, potentially shaping their perspectives. Although the study initially intended to use purposive sampling based on age and ethnicity, recruitment challenges contributed to the limited participation of individuals aged 16-17 years. As a result, 16-17-year-olds were not represented in the final sample, which meant that potential age-related differences in preferences and engagement with digital mental health interventions could not be explored. This is particularly relevant as the examined interventions have been evaluated with slightly younger adolescents (Bite Back for aged 12-18 years, Project YES for aged 11-17 years), which may have influenced how participants perceived and interacted with the content. Furthermore, as the interventions were designed to support young people experiencing symptoms of anxiety or depression, our participants’ generally low symptom levels may have affected how useful they found these interventions.

Participants were asked to use each intervention for approximately 30 minutes over a 2-week period. This decision was made to ensure consistency in the amount of time participants spent using each intervention, as Project YES was designed to be completed in a single 30-minute session, while also accommodating participants’ academic schedules. While this approach allowed participants to experience and compare both interventions within the same timeframe, it provided limited exposure to Bite Back, which is designed as a 6-week program (around 10 minutes per session), and may not have allowed participants sufficient time to fully engage with its features and content. Additionally, the short engagement period within a longer timeframe may have affected participants’ recall of content for both interventions, potentially influencing their perceptions and the depth of their feedback. To mitigate these limitations and aid recall, a think-aloud component was included, although it is acknowledged that the shortened engagement period may still have impacted the depth of participants’ experiences with the interventions.

### Implications for Development and Research

This study highlights the importance of involving young people in the co-design of digital mental health interventions to ensure content is age-appropriate, relatable, and engaging. In particular, future designs should consider the varying needs across different age ranges, as even small differences in age can affect how content is perceived and whether it feels relevant. Transparent communication about data use and the purpose of interventions should be clear and accessible while avoiding information overload. Future qualitative research could explore accessible and informative ways of communicating essential information, such as data use or the purpose of tasks, to build trust while maintaining engagement within digital mental health interventions.

### Conclusion

This study contributes to the growing body of knowledge by identifying 7 interconnected themes that could shape how young people engage with digital mental health interventions. These findings emphasize that young people value interventions that feel relevant to their age, culture, and stage of life, and that are transparent about their purpose, data use, and content. Even slight differences in age appeared to influence how appropriate the interventions felt to participants, highlighting the importance of involving young people in the design process to ensure interventions reflect their needs and preferences and clearly convey information that could support their engagement.

## Supplementary material

10.2196/84384 Multimedia Appendix 1 Topic guide.

## Abbreviations

ABC: Action Brings Change
CAMHS: Child and Adolescent Mental Health Services
CARE: Child and Adolescent Research in Emotion Lab
CATCH-IT: Competent Adulthood Transition with Cognitive Behavioral, Humanistic, and Interpersonal Training
CBT: cognitive behavioral therapy
COREQ: Consolidated Criteria for Reporting Qualitative Research
IHME: Institute for Health Metrics and Evaluation
OSF: Open Science Framework
RCADS: Revised Children’s Anxiety and Depression Scale
RCADS-25: Revised Children’s Anxiety and Depression Scale – 25 items
SWEMWBS: Short Warwick-Edinburgh Mental Wellbeing Scale
WEMWBS: Warwick-Edinburgh Mental Wellbeing Scale
YES: Youth Empowerment & Support
