# Supplementary material for: Adolescents’ Preferences, Experiences, and Views of 2 Online Programs for Anxiety and Depressive Symptoms: Qualitative Interview Study
Source: JMIR Pediatr Parent. 2026 Aug 18;9:e84384. doi: 10.2196/84384 (PMC13484819; doi:10.2196/84384)

SEMI-STRUCTURED TOPIC GUIDE (ETHICAL APPROVAL CODE: 21829)

Question	Prompts
1. Did you use your phone or laptop to access the interventions?	
1. Have you used any digital mental health platforms before this study?	• What were they?<br>• Could you tell me about your experience with them?<br>• How did you find out about these platforms?<br>• What did you hope to get out of using them?<br>• Was there anything you liked/disliked?<br>• Why not?
**Bite Back**	
2. How easy was it to start using Bite Back?	• Why?/Why not?<br>• Is there anything that could have made it easier to use it? Why?
3. What were your first thoughts when using Bite Back?	• What stood out to you? Why?<br>• Did anything surprise or confuse you?<br>• Did it feel welcoming?<br>• Which part wasn't as good as you expected? Why?
4. How easy or difficult was it to navigate through Bite Back interface?	• What were the specific parts that you found particularly hard to use?<br>• What were the specific parts that you found particularly easy to use?<br>• How could the navigation be improved?<br>• Did you like the use of a progress bar?<br>• Did you like having to unlock each week one by one?
5. Did you face any challenges while using Bite Back?	• What were they?<br>• How did you deal with them?
5. Did you get bored at any point while using Bite Back?	• Why/why not?<br>• How far in did you start to get bored?<br>• What would you say is the optimal amount of time to stay focused on one of these interventions?<br>• What could have made it more exciting/interesting to capture your engagement more?
6. How often did you use Bite Back over those 2 weeks?	• What influences how often you used it?<br>• When and where did you find yourself using it?
7. Did you find any of the features useful? – what features?	• Why?/Why not?
8. Were there any features you didn't find helpful?	• What made these features unhelpful?<br>• How did they influence your overall experience with Bite Back?<br>• Do you think they could have been cut without impacting the quality of the intervention?<br>• How else would you have done this?
9. How engaging was it to use Bite Back?	• Was there anything specific that was really engaging?<br>• Was there anything specific that lost your attention?<br>• Were there any features/visuals/interactions or content that helped keep you interested?
10. Was there anything that made it difficult for you to stay engaged with Bite Back?	• Were there times when you felt like stopping using it? Why?

	• What could have made it easier for you to stay engaged?
11. What did you think about the information shown in Bite Back?	• Did it feel informative?<br>• Did it feel age appropriate?<br>• Did it feel relevant?
12. Did your feelings or mood change while you were using Bite Back?	• What were the changes?
13. What did you think about the challenges/activities that it got you to do?	• Did you try the challenges?<br>• Why/why not?<br>• Was there anything that made it difficult to do them?<br>• Is there anything that would have made it easier to use these tips? Why?<br>• Would you have liked if it has saved your answers to go back and look at? Why?
14. What did think about seeing the information in a video?	• Why?<br>• Do you have a favourite way to see information, other than a video (such as diagrams, pictures, graphs, bullet points, audio?
15. Would you tell a friend to try Bite Back?	• Why?/Why not?<br>• What would you tell them about your experience?<br>• Are there specific reasons you think they would benefit from this platform?
16. Would you keep using Bite Back?	• Why?/Why not?<br>• How could it remind you/motivate you to keep using it?
17. What improvements would you suggest for Bite Back?	• Are there any features you think should be added or changed?
**Project Yes**	
18. Which activity/session did you decide to complete? (Project Personality, ABC, Care, or RISE)	• What made you choose it?<br>• Did you look at the other options? Why not?
18. Was it easy to start using Project YES?	• Why?/Why not?<br>• Is there anything that could have made it easier to use it? Why?
19. What were your first thoughts when using Project YES?	• What stood out to you? Why?<br>• Did anything surprise or confuse you?<br>• Did it feel welcoming?<br>• Which part wasn't as good as you expected? Why?
20. How easy or difficult was it to navigate through Project YES interface?	• What were the specific parts that you found particularly hard to use?<br>• What were the specific parts that you found particularly easy to use?<br>• How could the navigation be improved?<br>• Did you like not being able to see your progress or would you have liked a progress bar?<br>• Did you like being able to choose the activity you wanted to complete?
21. Did you face any challenges while using Project YES?	• What were they?<br>• How did you deal with them?
22. Did you get bored at any point while using Project YES?	• Why/why not?<br>• How far in did you start to get bored?

	• What would you say is the optimal amount of time to stay focused on one of these interventions?<br>• What could have made it more exciting/interesting to capture your engagement more?
22. How often did you use Project YES over those 2 weeks?	• What influenced how often you used it?<br>• When and where did you find yourself using it?
22. Did you find any of the features useful?	• Why?/Why not?
23. Were there any features you didn't find helpful?	• What made these features unhelpful?<br>• How did they influence your overall experience with Project YES?<br>• Do you think they could have been cut without impacting the quality of the intervention?<br>• How else would you have done this?
22. How engaging was it to use Project YES?	• Was there anything specific that was really engaging?<br>• Was there anything specific that lost your attention?<br>• Were there any features/visuals/interactions or content that helped keep you interested?
24. Was there anything that made it difficult for you to stay engaged with Project YES?	• Were there times when you felt like stopping using it? Why?<br>• What could have made it easier for you to stay engaged?
25. What did you think about the information shown in Project YES?	• Did it feel informative?<br>• Did it feel age appropriate?<br>• Did it feel relevant?
26. Did your feelings or mood change while you were using Project YES?	• What were the changes?
22. What did think about how the information was shown/formatted?	• Was it visually engaging?<br>• What would have liked to see more/less of?
27. Would you recommend Project YES to a friend?	• Why?/Why not?<br>• What would you tell them about your experience?<br>• Are there specific reasons you think they would benefit from this platform?
28. Would you keep using Project YES?	• Why?/Why not?<br>• How could it remind you/motivate you to keep using it?
29. What improvements would you suggest for Project YES?	• Are there any features you think should be added or changed?<br>• Why?
**Comparison**	
30. How could these platforms be more engaging?	•
31. Did you find any of the platforms more helpful?	• Why?/Why not?<br>• What specific aspects made it more helpful?
32. Do you see yourself continuing to use any of these platforms?	• Which one?<br>• Why?/Why not?
33. Is there anything else you'd like to say about Bite Back or Project YES?	

Examples:
Bite Back

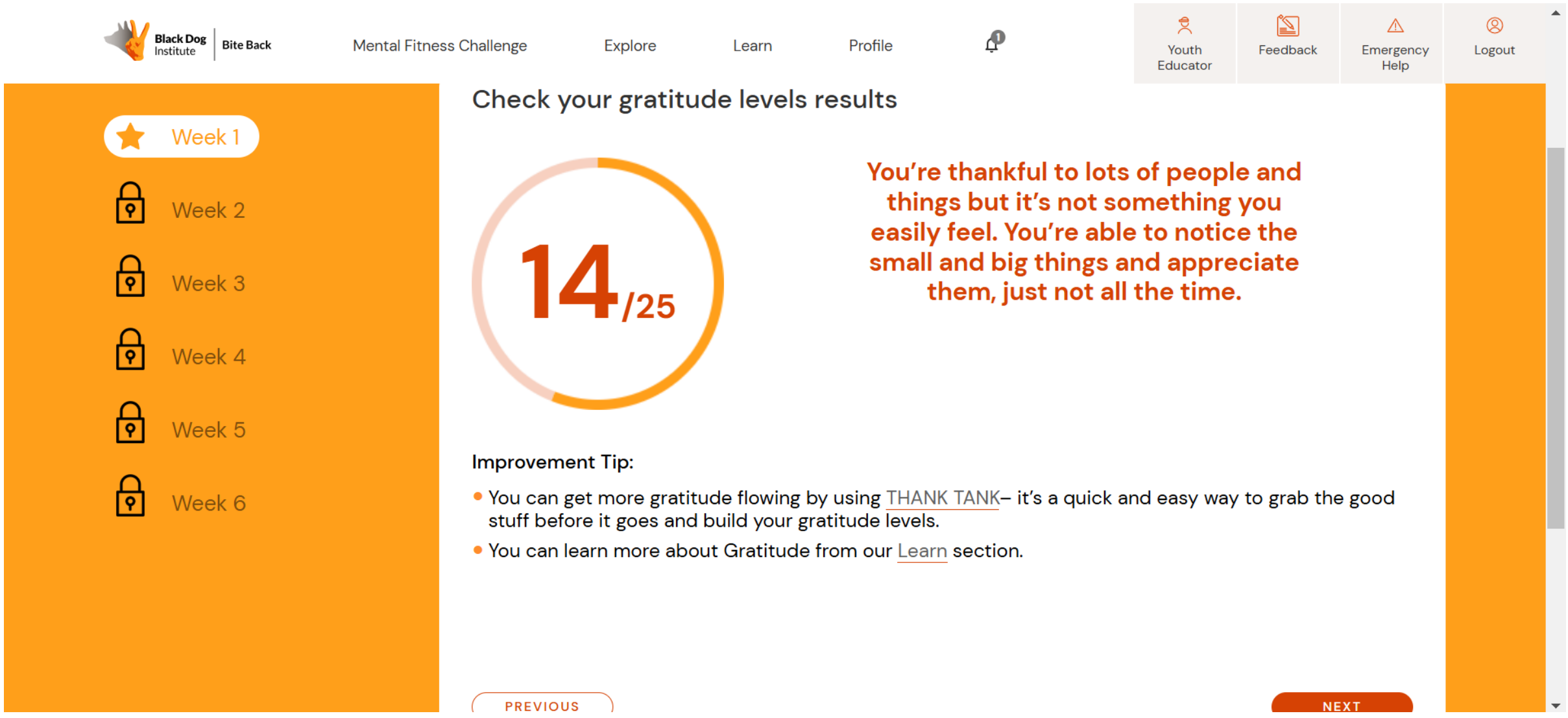

Project YES (Project Personality)

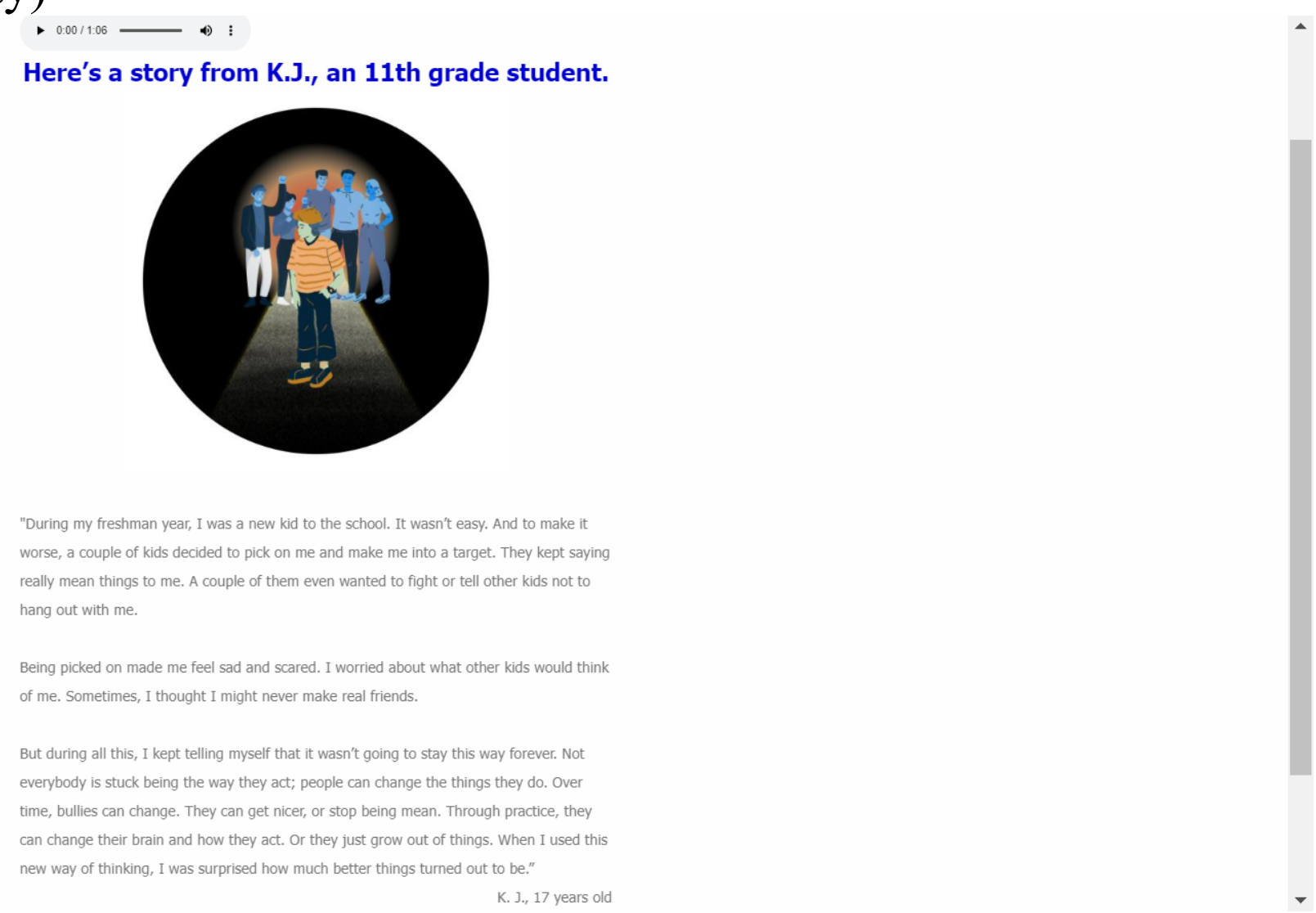

Supplement: Supplementary file 1 [file pediatrics-v9-e84384-s001.pdf]
